# Association Between Psychosocial Problems and Unhealthy Health Behavior Patterns Among Finnish Adolescents

**DOI:** 10.1007/s10578-020-00967-w

**Published:** 2020-02-12

**Authors:** Kaisa Marttila-Tornio, Heidi Ruotsalainen, Jouko Miettunen, Niko Männikkö, Maria Kääriäinen

**Affiliations:** 1 Research Unit of Nursing Science and Health Management, University of Oulu, Oulu, Finland; 2 Center for Life Course Health Research, University of Oulu, Oulu, Finland

**Keywords:** Adolescence, Childhood, Health behavior, Health behavior pattern, Psychosocial problems

## Abstract

The aim of the study was to investigate how psychosocial problems in childhood and adolescence associate with an unhealthy health behavior pattern among adolescents in Northern Finland. The study population consisted of 4350 participants, drawn from the Northern Finland Birth Cohort 1986 Study. Health behavior patterns were assessed in adolescence and psychosocial problems in childhood and adolescence. Logistic regression analyses were performed to determine the associations. Several psychosocial problems predicted greater likelihood of engaging in unhealthy health behavior pattern. Externalizing problems in childhood predicted greater likelihood of engaging in unhealthy behavior patterns for girls. For both genders, externalizing problems and inattention in adolescence were associated with unhealthy health behavior patterns. Boys and girls with externalizing problems both in childhood and adolescence had an increased risk of unhealthy patterns. Psychosocial problems contribute to unhealthy lifestyles and should therefore be acknowledged when designing and targeting health promotion strategies aimed at adolescents.

## Introduction

According to WHO, 60% of all deaths globally are caused by non-communicable diseases, and this figure is estimated to increase in the future. Non-communicable diseases are largely preventable by promoting healthy lifestyles that tackle unhealthy health behaviors [1]. Children and adolescents should be considered as an important target group for health promotion efforts since persistent health behaviors begin to solidify in adolescence [2] and thus create lifelong implications for individuals’ health and wellbeing [3]. Unhealthy health behaviors of adolescents increase the likelihood of poorer health both when young and in later years, making adolescents’ unhealthy lifestyles a global concern [4].

Health behaviors are identified as overt behavioral patterns, actions, and habits that relate to health maintenance, to health restoration, and to health improvement [5]. Health behaviors, unhealthy behaviors included, do not occur in isolation but rather cluster together. To inform a more comprehensive approach to adolescents’ health behaviors and health promotion procedures, it is important to examine *patterns* of health behaviors instead of just concentrating on individual health behaviors as has hitherto been largely the case [6–8]. Health behaviors cluster in complex ways, a fact which has been shown in many previous studies. For example, a review by Leech et al. [8] found that diet, physical activity, and sedentary behavior cluster in both healthy and unhealthy ways, although co-occurrence of both healthy and unhealthy behaviors was also identified.

Mental health is an integral part of overall health and wellbeing. Mental health problems may begin to develop early in childhood and continue into adulthood [9]. Therefore, it is important to address mental health problems and their potential adverse effects on lifestyle as early as possible. Determinants of mental health include multiple psychological and social factors [10]. Psychological factors include individual attributes that influence mental states. In contrast, social factors are general factors operating at the higher level of human society; these forces are concerned with social structures and processes that impinge on the individual’s mindset. [11] According to WHO, the mental health of children and adolescents is globally less than optimal. Indeed, an estimated 10–20% of children and adolescents experience mental health conditions. Mental health problems are a major source of illness and disability among adolescents and are commonly experienced as internalizing (depression and/or anxiety) and externalizing (conduct disorders as well as hyperactivity/inattention disorders) disorders [12].

Several studies have shown that internalizing, externalizing, and inattention problems are associated with multiple individual unhealthy health behaviors among children and adolescents. For example, fewer internalizing problems have been reported among children who are active in sports [13]. Depression or poor mental health were found to be linked to an unhealthy diet in a systematic review by Khalid et al. [14]. Also, according to two other systematic reviews, children’s and adolescents’ inattention problems are connected to higher screen time [15] and unhealthy diet [16]. A previous study conducted of the Northern Finland Birth Cohort 1986 (NFBC 1986) showed externalizing problems in childhood to predict adolescent cigarette smoking and use of other substances [17]. Externalizing problems in childhood also predict increased alcohol use in adolescence according to large longitudinal studies in the US and the UK [18, 19]. Similar results were found in a study of the psychosocial problems and alcohol consumption of adolescents [20]. A link between smoking and internalizing problems, externalizing problems as well as hyperactivity has also been reported in a large sample of European adolescents [21].

Previous studies have clearly shown that psychosocial problems affect health behaviors, but it is also recognized that health behaviors affect psychosocial problems: a population-based research of 10- to 11-year-old children reported that also unhealthy health behaviors, such as low physical activity and high use of computers and video games, in childhood are connected to externalizing and internalizing problems during adolescence [22]. These results reveal the multidimensional interplay between health behaviors and psychosocial health which should be taken into consideration in health promotion programs aimed at children and adolescents.

In the development and targeting of effective health promotion strategies that can prevent negative health outcomes, it is crucial to understand the factors that contribute to unhealthy health behavior patterns in adolescence [3]. Recognizing these factors makes it possible to find out if the risk of developing lifestyle diseases accumulates among specific subgroups of adolescents [23]. Instead of looking at health behavior patterns, previous research has mostly focused on investigating the association between psychosocial factors and individual health behaviors. Given the clustering of health behaviors it is also important to investigate the factors affecting them. The aim of this study is to investigate how psychosocial problems in childhood and adolescence predict unhealthy health behavior patterns at the age of 16.

## Methods

### Data

The study population comprised a prospective mother–child birth cohort called the Northern Finland Birth Cohort. NFBC 1986 originally included 9432 children born alive in the two most northern provinces in Finland (Oulu and Lapland) with an expected date of birth between 1st July 1985 and 30th June 1986. The research was approved by the ethical committee of the Northern Ostrobothnia Hospital District in Oulu, Finland. Participation was voluntary and based on informed consent (https://www.oulu.fi/nfbc/node/44315).

This study is based on the two large comprehensive follow-up studies of the NFBC 1986 conducted so far. The 8-year stage follow-up study of the cohort was conducted in the spring of the children’s first school year (either 1993 or 1994). Teachers of the participants completed questionnaires that included items relating to psychosocial problems. A total number of 8525 participants’ questionnaires were completed (response rate 91.7%). In the years 2000–2001 16-year-old cohort members completed a follow-up postal survey that included sections on various health-related behaviors and psychosocial problems. Questionnaire data was received from 7182 adolescents (response rate 77.9%). This questionnaire was used to identify participants’ health behavior patterns in adolescence.

The final sample of this study consists of 4305 (boys = 2003, girls = 2303) participants, who answered questions on all of the health behaviors included in the study (physical activity, screen time, cigarette smoking, alcohol use, and diet). The data of these 4305 participants was used to analyze the association between psychosocial problems in childhood and adolescence and health behavior patterns at the age of 16.

## Measures

### Health Behavior Patterns

Health behavior patterns among 15- to 16-year-old boys and girls participating in the NFBC 1986 Study have been identified previously [24]. In that particular study, participants completed a questionnaire evaluating the following health behaviors: physical activity, screen time, cigarette smoking, alcohol use, and diet. Participants were asked to indicate the number of hours per week devoted to vigorous exercise outside of school hours. Response options varied from “1 = not at all” to “6 = 7 h a week or more”. The variable was then categorized into the following categories: 1 = not at all to about an hour per week; 2 = 2–3 h per week; and 3 = about 4–7 h per week.

Screen time was ascertained with the following questions: “On average, how many hours a day do you watch TV outside of school hours?”, and “On average, how many hours a day do you play or use the computer and/or video games outside of school time?” A sum variable based on the hours reported was created and then categorized into the following categories: less than or equal to 2 h per day and more than 2 h per day.

Cigarette smoking was assessed with the question: “At the present time, do you smoke cigarettes?” Response categories ranged from “1 = not at all” to “6 = 7 days per week”. The variable was then categorized into the following categories: 1 = not at all; 2 = occasionally to 2–4 days a week; and 3 = 5–7 days a week.

Information on alcohol use was evaluated with the question: “Have you ever drank or do you still—even occasionally—drink some alcoholic beverages?” Response categories ranged from “1 = never” to “6 = at least once a week or more”. The variable was then categorized into three categories: 1 = never to have tasted but do not presently consume; 2 = consume casually to consume about once a month; and 3 = consume 2–3 times a month to consume at least once a week or more.

Participants were asked to indicate how often they eat each of the following sugary foods: cake and cookies, ice cream, sugary beverages, chocolate, and sweets. Participants also indicated how often they eat each of the following fast foods: French fries or fried potatoes, hamburgers or pizza, and chips. Response options for each question ranged from “1 = less than once a month or not at all” to “7 = once a day or more”, and a sum variable was created based on the responses. Fruit, vegetable, and berry intake was assessed with questions indicating how many times during the past week participants ate the following foods: uncooked vegetables, uncooked fruit or fruit salad, and berries. Response categories for each question ranged from “1 = not at all” to “4 = 6–7 times per week”. A sum variable was then created based on the responses.

A cluster analysis of previous health behaviors revealed two health behavior patterns for both genders. The healthy health behavior pattern denoted as “Healthy Lifestyle” consists of the most positive scores in terms of all the included health behaviors. The unhealthy health behavior pattern summarized as “Unhealthy Lifestyle” is characterized by unhealthy behavior in terms of all the health behaviors. The majority of adolescents (61% of the boys; n = 1215 and 58% of the girls; n = 1340) were grouped in the “Healthy Lifestyle” cluster. “Unhealthy Lifestyle” consisted of a slightly higher proportion of the girls (42%; n = 962) than boys (39%; n = 788).

### Psychosocial Problems

The Rutter Children’s Behavioral Questionnaire, Rutter B2 Scale, for teachers [25] was used to assess psychosocial problems at the age of 8 (children’s first school year). It is widely used and valid in a Finnish sample of 8-year-old children [26]. The Rutter B2 Scale comprises 26 items with the answer alternatives “fits fine” (2 points); “fits partly” (1 point); or “does not fit” (0 points). Three subscales can be formed from the items: internalizing (the sum of items—often worried, miserable, fearful, and tears on arrival at school), externalizing (the sum of items—destructive, fights, disobedient, lies, steals, and bullies), and hyperactivity (the sum of items—restless, squirmy and fidgety, and poor concentration). Scores on the questions were added together to obtain a summary score for each subscale. Participants were dichotomized as normal range and clinical range groups with the recommended cut-off limit for clinically significant problems at the 90th percentile [27]. In the present study this meant ≥ 2 points (both genders) for internalizing problems, ≥ 4 points (boys) or ≥ 2 (girls) points for externalizing problems, and ≥ 4 points (boys) or ≥ 3 (girls) points for hyperactivity. If there was one missing item in a subscale, the missing item was imputed with the mean item score for that individual in the scale in question. Those with more than one missing item were excluded. In the current sample, Cronbach’s α for teachers’ ratings was 0.68 for internalizing, 0.84 for externalizing problems, and 0.89 for hyperactivity.

A Youth Self-Report (YSR) questionnaire was used to measure psychosocial problems at the age of 16. The validity and reliability of the YSR have proven to be good [27]. The questionnaire included 105 items with answer alternatives “applies very well or often” (2 points), “applies somewhat or occasionally” (1 point), or “does not apply” (0 points). Based on these a sum score can be calculated for eight subscales: anxious/depressed, withdrawn/depressed, somatic complaints, social problems, thought problems, attention problems, rule-breaking behavior, and aggressive behavior. Anxious/depressed (e.g. I cry a lot), withdrawn/depressed (e.g. I lack energy), and somatic complaints (e.g. I have headaches) are regarded as related to internalizing problems (total of 30 items). Rule-breaking behavior (e.g. I drink alcohol) and aggressive behavior (e.g. I scream a lot) are indicators of externalizing problems (total of 29 items). Attention problems (e.g. I cannot sit still) were considered as inattention problems (total of seven items). Social problems and thought problems were not included in the analyses in this study.

The items were summed and scored on internalizing (score range 0–60), externalizing (score range 0–58), and attention (score range 0–14) problems. To provide a common scale for comparisons with the Rutter B2 Scale, adolescents were dichotomized as normal range and clinical range categories. The recommended cut-off limit of the 82nd percentile for clinically significant problems was used [27]. In the present study this meant ≥ 10 points (boys) or ≥ 18 points (girls) for internalizing problems, ≥ 14 points (boys) or ≥ 16 (girls) points for externalizing problems, and ≥ 5 points (boys) or ≥ 6 (girls) points for inattention. Those with equal or over three (internalizing and externalizing problems) or over one (inattention problems) missing items were excluded from the analyses. If there were less than three (internalizing and externalizing problems) or only one missing item (inattention problems), the missing item was imputed with the mean item score for that individual in the scale in question. The alpha coefficient was 0.87 for internalizing problems, 0.87 for externalizing problems, and 0.64 for inattention.

We also calculated a score from both scales (Rutter B2 Scale and YSR) to indicate possible long-term externalizing problems, internalizing problems, and hyperactivity/inattention problems from clinically significant symptoms at age 8 and 16. For each of the psychosocial problems scores were categorized into two levels: problems in childhood as well as in adolescence (possible long-term problems), and other (no problems at all, or problems only in childhood or adolescence).

### Other Factors

Parents’ education and family structure were used as covariates in the adjusted analyses. When the participants were 8 years old their parents reported their family structure. At the age of 16, parents of the participants reported their family structure and the highest occupational education level. We specified family structure as a combination of childhood and adolescence family structure, and it was categorized as both parents living with the participant (intact families) and other (non-intact families). Parents’ education level when the adolescent was aged 16 was categorized as high, medium, low, and other/uncompleted.

### Data Analysis

All data analyses and tests were performed using SPSS for Windows (version 25.0; IBM, Armonk, NY, USA). Previous studies have indicated that adolescents’ health behavior patterns differ according to gender [8], hence all analyses in our study were also performed separately for boys and girls. Boys’ and girls’ health behavior patterns in adolescence were identified using k-means non-hierarchical cluster analysis. To minimize the influence of variables with larger absolute measurement ranges relative to those with smaller ranges, cluster variables were first standardized. Several cluster solutions were tested. The most reliable cluster solution was chosen based on the kappa coefficient tests between the original data set and subsamples randomly taken from it. For both genders, a two-cluster solution turned out to be most reliable. A more thorough explanation about the cluster analysis can be found elsewhere [24].

Data from childhood and adolescence were used in separate analyses. Analyses concerning possible long-term psychosocial variables were also performed separately. Descriptive statistics were computed for the data set to describe the sample by psychosocial problems. Chi-square tests were used to examine differences in clusters according to psychosocial problems. Cross-tabulation and chi‐squared tests were used to examine the associations between health behavior patterns in adolescence and psychosocial problems as well as covariates.

Logistic regression analyses (unadjusted and adjusted) were performed to determine the associations between different health behaviors and psychosocial problems. Unadjusted analyses were performed first for each of the psychosocial variables. In the adjusted analyses, the association between psychosocial problems and health behavior patterns was evaluated in a regression model in which all variables were incorporated simultaneously. In the adjusted analyses, potential associations were controlled for confounding by parents’ occupational education and family structure. Odds ratios with their 95% confidence intervals (CIs) are presented. The “Unhealthy Lifestyle”, representing a negative combination of unhealthy health behavior in terms of all the health behaviors, was the reference category. Statistical significance (p-value) was established as p ≤ 0.05.

## Results

Statistics describing the sample (n = 4305) by psychosocial problems are presented in Table 1. Overall, girls in the unhealthy health behavior cluster had significantly more internalizing (17.7 vs. 14.4%, p < 0.05), externalizing (13.7 vs. 8.3%, p < 0.05), and hyperactivity (10.6 vs. 6.4%, p < 0.05) problems in childhood than girls in the healthy health behavior pattern. Boys in the unhealthy health behavior cluster had significantly more hyperactivity problems (12.9 vs. 9.7%, p < 0.05) in childhood than boys in the healthy health behavior cluster. In adolescence, girls in the unhealthy health behavior cluster had significantly more internalizing (27.2 vs. 19.8%, p < 0.05), externalizing (41.6 vs. 16.2%, p < 0.05) as well as inattention (43.7 vs. 24.3%, p < 0.05) problems. Correspondingly boys in the unhealthy health behavior cluster had significantly more internalizing (27.8 vs. 21.4%, p < 0.05), externalizing (42.7 vs. 13.5%, p < 0.05) as well as inattention (40.0 vs. 24.0%, p < 0.05) problems.

**Table 1:** Descriptive statistics (frequency and percentage) of included variables in childhood and adolescence from the Northern Finland Birth Cohort 1986 (n = 4305)

	Boys n = 2003				Girls n = 2302			
	Healthy lifestyle n = 1215	Unhealthy lifestyle n = 788	Total n = 2003	p-value	Healthy lifestyle n = 1340	Unhealthy lifestyle n = 962	Total n = 2302	p-value
	n (%)	n (%)	n (%)		n (%)	n (%)	n (%)	
Childhood								
Internalizing	179 (14.7)	131 (16.6)	310 (15.5)	0.23	180 (14.4)	159 (17.7)	339 (14.7)	0.041*
Externalizing	125 (10.3)	100 (12.7)	225 (11.2)	0.09*	104 (8.3)	123 (13.7)	227 (9.9)	< 0.001*
Hyperactivity	118 (9.7)	102 (12.9)	220 (11.0)	0.023*	80 (6.4)	95 (10.6)	172 (7.5)	0.001*
Adolescence								
Internalizing	260 (21.4)	219 (27.8)	479 (23.9)	0.001*	263 (19.8)	259 (27.2)	522 (22.7)	< 0.001*
Externalizing	164 (13.5)	332 (42.7)	496 (24.8)	< 0.001*	216 (16.2)	399 (41.6)	616 (26.7)	< 0.001*
Inattention	292 (24.0)	307 (40.0)	599 (29.9)	< 0.001*	325 (24.3)	417 (43.7)	742 (32.2)	< 0.001*

*Statistically significant differences (χ^2^ tests, p < 0.05)

### Childhood Psychosocial Problems

Results of the regression analyses for childhood are shown in Table 2. In the unadjusted analyses, internalizing problems (OR 1.28, 95% CI 1.01–1.61), externalizing problems (OR 1.75, 95% CI 1.33–2.31), and hyperactivity (OR 1.73, 95% CI 1.27–2.37) in childhood significantly predicted a greater likelihood of engaging in unhealthy health behavior patterns in adolescence for girls. In the adjusted model only externalizing problems (OR 1.56, 95% CI 1.03–2.36) were significantly associated with unhealthy lifestyle. For boys, only hyperactivity (OR 1.40, 95% CI 1.05–1.86) was significantly associated with unhealthy lifestyle in the unadjusted model, whereas the other associations were insignificant. In the adjusted model none of the psychosocial problems were significantly associated with unhealthy lifestyle.

**Table 2:** Odds ratios with 95% confidence intervals of the associations between psychosocial problems in childhood and unhealthy health behavior pattern in adolescence in Northern Finland Birth Cohort 1986 follow-up survey in 1993–1994 (n = 4305)

Childhood	Boys Unhealthy lifestyle (adolescence) n = 788				Girls Unhealthy lifestyle (adolescence) n = 962			
	Unadjusted		Adjusted		Unadjusted		Adjusted	
	OR	95% CI	OR	95% CI	OR	95% CI	OR	95% CI
Internalizing problems	1.17	0.91–1.50	0.92	0.63–1.34	1.28*	1.01–1.61	1.15	0.83–1.60
Externalizing problems	1.27	0.96–1.69	1.03	0.65–1.64	1.75*	1.33–2.31	1.56*	1.03–2.36
Hyperactivity	1.40*	1.05–1.86	1.08	0.68–1.73	1.73*	1.27–2.37	1.18	0.74–1.91

Unhealthy cluster is the reference group in logistic regression

OR odds ratio, 95% CI confidence interval

All results in the adjusted model were adjusted for parents’ occupational education and family structure

*p < 0.05

### Adolescence Psychosocial Problems

Results of adolescence regression analyses are presented in Table 3. For both genders, internalizing problems (OR 1.44, 95% CI 1.17–1.77 for boys, OR 1.52, 95% CI 1.25–1.84 for girls), externalizing problems (OR 4.76, 95% CI 3.83–5.92 for boys, OR 3.70, 95% CI 3.05–4.50 for girls), as well as inattention (OR 2.04, 95% CI 1.68–2.48 for boys, OR 2.41, 95% CI 2.02–2.88 for girls) in adolescence were significantly associated with unhealthy health behavior pattern in the unadjusted model, whereas in the adjusted model only externalizing problems (OR 4.33, 95% CI 3.13–5.99 for boys, OR 3.17, 95% CI 2.38–4.23 for girls) and inattention (OR 1.46, 95% CI 1.07–1.99 for boys, OR 1.66, 95% CI 1.24–2.21 for girls) had a significant association with unhealthy lifestyle.

**Table 3:** Odds ratios with 95% confidence intervals of the associations between psychosocial problems in adolescence and unhealthy health behavior pattern in adolescence in Northern Finland Birth Cohort 1986 follow-up survey in 2000–2001

Adolescence	Boys Unhealthy lifestyle (adolescence) n = 788				Girls Unhealthy lifestyle (adolescence) n = 962			
	Unadjusted		Adjusted		Unadjusted		Adjusted	
	OR	95% CI	OR	95% CI	OR	95% CI	OR	95% CI
Internalizing problems	1.44*	1.17–1.77	0.77	0.55–1.07	1.52*	1.25–1.84	0.86	0.63–1.18
Externalizing problems	4.76*	3.83–5.92	4.33*	3.13–5.99	3.70*	3.05–4.50	3.17*	2.38–4.23
Inattention	2.04*	1.68–2.48	1.46*	1.07–1.99	2.41*	2.02–2.88	1.66*	1.24–2.21

(n = 4305)

Unhealthy cluster is the reference group in logistic regression

OR odds ratio, 95% CI confidence interval

All results in the adjusted model were adjusted for parents’ occupational education and family structure

*p < 0.05

### Long-Term Psychosocial Problems

When studying those with long-term problems, boys who had externalizing problems both in childhood and adolescence had an increased risk of unhealthy health behavior patterns in adolescence in the unadjusted (OR 3.33, 95% CI 2.02–5.47) and adjusted models (OR 2.98, 95% CI 1.41–6.28). Internalizing problems or hyperactivity/inattention both in childhood and adolescence were not associated with unhealthy health behavior pattern. Girls who had internalizing (OR 1.62, 95% CI 1.07–2.47), externalizing (OR 4.26, 95% CI 2.54–7.13), or hyperactivity/inattention (OR 2.86, 95% CI 1.79–4.59) problems both in childhood and adolescence had an increased risk for unhealthy health behavior pattern in the unadjusted model. In the adjusted model, only girls who had externalizing problems both in childhood and adolescence (OR 3.46, 95% CI 1.60–7.45) had an increased risk for unhealthy health behavior pattern. These results are presented in Table 4.

**Table 4:** Odds ratios with 95% confidence intervals of the associations between long-term psychosocial problems (both in childhood and adolescence) and unhealthy health behavior pattern in adolescence in Northern Finland Birth Cohort 1986 (n = 4305)

Childhood and adolescence	Boys Unhealthy lifestyle (adolescence) n = 788				Girls Unhealthy lifestyle (adolescence) n = 962			
	Unadjusted		Adjusted		Unadjusted		Adjusted	
	OR	95% CI	OR	95% CI	OR	95% CI	OR	95% CI
Internalizing problems	1.14	0.71–1.81	0.77	0.35–1.70	1.62*	1.07–2.47	0.48	0.81–2.71
Externalizing problems	3.33*	2.02–5.47	2.98*	1.41–6.28	4.26*	2.54–7.13	3.46*	1.60–7.45
Hyperactivity/inattention	1.41	0.91–2.18	1.29	0.66–2.53	2.86*	1.79–4.59	1.85	0.92–3.74

Unhealthy cluster is the reference group in logistic regression

OR odds ratio, 95% CI confidence interval

All results in the adjusted model were adjusted for parents’ occupational education and family structure

*p < 0.05

## Discussion

This study provides a comprehensive approach to studying adolescents’ health behaviors and to the psychosocial factors affecting them. We examined how psychosocial problems predict health behavior patterns in a large sample of adolescents aged 16. For girls, externalizing problems in childhood predicted a greater likelihood of engaging in unhealthy health behavior pattern in adolescence. For boys, no significant associations were found. Externalizing problems and inattention in adolescence were associated with unhealthy health behavior patterns for both genders. Boys and girls with externalizing problems both in childhood and adolescence had an increased risk of unhealthy patterns.

Results of our study suggest that for girls externalizing problems in childhood predicts a greater likelihood of engaging in unhealthy health behavior patterns in adolescence. The link between externalizing problems in childhood and unhealthy health behaviors in adolescence has also been found in previous studies, though they have mainly focused on the relationship between psychosocial problems and smoking and/or substance use. In a population-based study by Dick et al. [28] children with internalizing and externalizing difficulties were found to have elevated rates of alcohol problems in adolescence. Externalizing problems also had the strongest association with subsequent tobacco use in a prospective birth cohort study that examined whether child and adolescent psychopathology predicts subsequent tobacco use at adolescence and in young adulthood [29]. In addition, results of a longitudinal, community-based study of twins suggest that for both genders externalizing problems predicts not only smoking and alcohol use but also cannabis use in adolescence [30].

According to previous research, externalizing problems in childhood is not only linked to health behaviors but also to poor health outcomes in adolescence. Findings from two large prospective birth cohort studies suggest that externalizing behavior in early childhood is followed by increases in body mass index in later childhood [31, 32]. A systematic review also found an association between childhood and adolescent externalizing problems and increased risk of cardiovascular diseases and type-2 diabetes [33]. These results might be explained by the fact that externalizing problems in childhood increases the likelihood of engaging in unhealthy health behaviors, and unhealthy health behavior patterns in turn may increase the likelihood of poor health outcomes. In order to prevent unhealthy health behaviors and poor health outcomes, an early identification of the determinants affecting them is needed.

We found externalizing problems in adolescence to be associated with unhealthy health behavior patterns for both genders. Also, long-term externalizing problems both in childhood and adolescence increased the risk of unhealthy patterns. Externalizing problems’ connection with unhealthy health behaviors has been showed in previous research, but it should be noted that a link between internalizing problems and some unhealthy health behaviors has also been found. For example, positive association between internalizing, externalizing, and hyperactivity problems and smoking was found in a large representative sample of European youths [21]. Also, a link between both internalizing and externalizing problems and inactivity was identified in a large and representative population survey of 11- to 16-year-old adolescents [34]. A recent systematic review also revealed a connection between externalizing problems and binge drinking among adolescents and young adults [20].

According to our research, inattention is also connected to unhealthy health behavior patterns. In line with our results, prior studies have reported an association between attention problems and unhealthy health behaviors, although this earlier work has particularly focused on the relationship between ADHD and health behaviors. One review found a connection between hyperactivity/inattention problems and high levels of screen time [15], while another systematic literature review found a link between ADHD and unhealthy diet [16]. According to a population-based study of 5- to 17-year-old US children, multiple unhealthy health behaviors—such as smoking, high television/video exposure, and low participation in organized sports—are also associated with ADHD [35]. Results from another US population-based study support the finding that children and adolescents with ADHD are less likely to engage in regular vigorous physical activity and organized sports [36]. Co-occurrence of multiple unhealthy health behaviors (such as snacking and sedentary time) has also been previously identified among children with ADHD [37].

Few studies that have investigated the link between the psychosocial differences and health behavior patterns in adolescence confirm our results that psychosocial problems mostly associate with unhealthy health behavior patterns. Veloso et al. [7] found that unhealthy psychosocial factors, such as poor wellness and health perception, low self-regulation, motivation, and body (dis)satisfaction, associate with unhealthier health behavior patterns. Accordingly, a more favorable psychosocial pattern predicted the healthiest health behavior pattern identified. Berlin et al. [38] found higher rates of socio-emotional protective factors (locus of control, socio-emotional functioning) and lower rates of risk factors (internalizing scores) among adolescents in the healthiest health behavior pattern. Adolescents with unhealthier health behavior patterns reported higher internalizing problems and lower self-concept. In the study by Iannotti and Wang [39] more frequent depressive symptoms were found among adolescents in the unhealthy health behavior cluster. Adolescents in the healthy health behavior pattern consistently reported higher quality of life and lower depressive symptoms. The findings of our study revealed more adolescents in the unhealthy health behavior cluster had internalizing problems (including depressive symptoms), but in regression analyses internalizing problems were significantly associated with unhealthy health behavior patterns only in the unadjusted models. The difference may be explained by the fact that in the adjusted model the confounding effects of psychosocial problems on each other as well as the confounding effects of parents’ education and family structure were considered.

Our results verify the clustering of adolescents’ unhealthy health behaviors and their association with psychosocial problems. This is consistent with the study of de Looze et al. [40] that examined cross-national consistencies in the clustering of adolescent risk behaviors and their psychosocial correlates. Adolescents’ risk behaviors were found to co-occur and share common psychosocial risk factors across countries. It is also recognized that adolescents’ risk of mental health problems increases if they exhibit more than one unhealthy behavior [41], something which verifies the importance of evaluating the association of psychosocial problems with multiple health behaviors and health behavior patterns.

A major strength of the present study is the use of data derived from a large, longitudinal cohort. The NFBC 1986 population can be considered as representative of Finnish adolescents, and the population-based nature of the sample reduces the likelihood of selection bias. Our study adds valuable information about the association between psychosocial factors and health behavior patterns, where previous studies have mostly concentrated on investigating the association of psychosocial factors with single health behaviors. Our approach enables us to form a better general view of the interplay among psychosocial problems and health behaviors which can be utilized in health promotion planning.

Against the foregoing strengths, a few limitations of this study should also be taken into account. The psychosocial problems in childhood were assessed by teachers of the then 8-year-old children, whereas at the age of 16 the assessment was done by the adolescents themselves. Therefore, differences in the assessments may be due to the vagaries of different data reporters, rather than down to changes in the levels of problem behavior at the ages of 8 and 16. There are also uncertainties in how well the teachers were able to assess the children’s psychosocial factors fully, as their assessment was mainly based on behavior in the school environment, not in other environments or in the children’s spare time. The information on health behaviors and psychosocial factors in adolescence was based on self-reporting, and self-reports have well-known problems: respondents tend to preferentially give responses which they consider desirable, and under- or over-reporting may have occurred. One limitation of the study is that the long-term psychosocial variables we created reflect participants’ psychosocial problems only at the age of 8 and 16, so the variables do not truly capture long-term data as there is no information on the psychosocial problems in between these two ages. Although NFBC 1986 is a longitudinal research program, we did not use longitudinal analyses in our study as it would have required more frequent follow-ups than have been done in NFBC 1986 so far.

## Summary

This study provides new insights in the field of psychosocial problems and unhealthy behavior patterns in adolescents. Since health behaviors cluster, it is important to focus not only on individual health behaviors, but also on health behavior patterns in order to have the most comprehensive information available on adolescents’ health behaviors and the factors affecting them. Results of this study suggest that psychosocial problems contribute to unhealthy lifestyles and should therefore be acknowledged when targeting health promotion strategies aimed at adolescents. In order to plan and execute more effective health promotion strategies it is important to identify children and adolescents who are at risk of developing unhealthy lifestyles and are thus at risk from a variety of lifestyle-related diseases. Identifying and targeting these subgroups as early as possible in their lives can protect them from the development of unhealthy lifestyles.
